# Diagnostic Approach and Therapeutic Management of Spontaneous Esophageal Perforation (Boerhaave’s Syndrome): A Systematic Review and Individual Patient-Level Pooled Analysis

**DOI:** 10.3390/jcm15187032

**Published:** 2026-09-11

**Authors:** Evgenia Mela, Maximos Frountzas, Nefeli Tomara, Dimitrios Damaskos, Nektaria Karangeli, Adam Mylonakis, Markos Despotidis, Aristeidis Sourgiadakis, Nikoletta Dimitriou, George Tribonias, Dimitrios Schizas

**Affiliations:** 1 First Department of Surgery, National and Kapodistrian University of Athens, Laikon General Hospital, 11527 Athens, Greece; nekkarangeli@gmail.com (N.K.); adam.mylonakis@gmail.com (A.M.); markosd1995@yahoo.gr (M.D.); aristeidis.sou@gmail.com (A.S.); nicole-demetriou@outlook.com (N.D.); schizasad@gmail.com (D.S.); 2 Department of Colorectal Surgery, Royal Marsden Hospital, NHS Foundation Trust, London SW3 6JJ, UK; froumax@hotmail.com; 3 Second Propaedeutic Department of Surgery, National and Kapodistrian University of Athens, Laikon General Hospital, 11527 Athens, Greece; neftomara@gmail.com; 4 General and Upper GI Surgical Unit, Royal Infirmary of Edinburgh, Edinburgh EH16 4SA, UK; dimitris.damaskos@gmail.com; 5 Department of Gastroenterology, Red Cross Hospital, 11526 Athens, Greece; gtrimponias@gmail.com

**Keywords:** Boerhaave’s syndrome, spontaneous esophageal perforation, endoscopic therapy, surgical management

## Abstract

**Background/Objectives:** Boerhaave’s syndrome (BS) is a rare, life-threatening condition associated with high mortality. This systematic review aimed to summarize the clinical presentation, management, and outcomes of adult patients with BS. **Methods:** PubMed and Scopus were searched through 12 June 2026 for case reports and case series reporting individual patient-level data. Demographic, clinical, diagnostic, therapeutic, and outcome data were extracted. Risk of bias was assessed using the Joanna Briggs Institute Critical Appraisal Checklist, and factors associated with in-hospital mortality were explored using logistic regression. **Results:** A total of 427 studies comprising 694 patients were included. Mean age was 57.1 years and 523 patients (80.6%) were male. Vomiting (79.5%) and chest pain (60.7%) were the most common symptoms, while 263 patients (67.6%) met SIRS criteria at diagnosis. The distal esophagus was involved in 83.5% of cases, and computed tomography was the predominant diagnostic modality (55.5%). Surgery was the most common first-line treatment (62.8%). In-hospital and 30-day mortality were 18.2% and 15.4%, respectively. In exploratory analyses, increasing age and SIRS were associated with in-hospital mortality. Risk of bias assessment identified methodological concerns across studies predominantly due to selective and incomplete outcome reporting. Certainty of evidence for in-hospital mortality was very low according to GRADE. **Conclusions:** BS remains associated with substantial morbidity and mortality. Surgery remains the most frequently applied treatment, while conservative and endoscopic strategies are used in selected patients. Case-based evidence precludes reliable comparative assessment of treatment effectiveness. The association between SIRS and mortality remains hypothesis-generating.

## 1. Introduction

Boerhaave’s syndrome (BS) is a rare but life-threatening clinical entity, characterized by spontaneous esophageal perforation resulting from sudden distal esophageal pressurization, most frequently precipitated by forceful vomiting, in the context of negative intrathoracic pressure [1,2]. Although it was first described in 1724 by Hermann Boerhaave, successful surgical management was not reported until more than two centuries later by Barrett et al. [3,4]. BS accounts for approximately 15% of all esophageal perforations, with an age-standardized incidence of 0.31 cases per 100.000 population annually [5]. Despite substantial improvements in diagnostic and therapeutic management, mortality rates remain considerable, ranging from 15% to 50%, due to mediastinal contamination and sepsis development [1,5].

The principles of treatment include hemodynamic support, definitive closure of the esophageal defect and adequate drainage of pleural collections. Although surgical repair has traditionally been the mainstay of treatment, the therapeutic spectrum has expanded considerably with advances in interventional endoscopy [1,6]. Endoscopic approaches, comprising self-expandable metal stent placement and endoscopic vacuum therapy (EVT), provide less invasive, organ-preserving options in appropriately selected patients [6,7,8]. A recent multinational retrospective cohort study found no significant differences in in-hospital and 90-day mortality between endoscopic and surgical approaches, although treatment selection depends strongly on clinical presentation [9].

Nevertheless, the existing evidence for BS largely derives from retrospective single-center series, while larger cohorts frequently combine spontaneous perforation with other etiologies of esophageal injury. Consequently, uncertainty persists regarding the optimal management of BS and guideline recommendations rely on low-certainty evidence [10]. This heterogeneity limits the comparability of reported outcomes and complicates clinical decision-making. This systematic review and individual patient-level pooled analysis aims to synthesize current evidence on the clinical presentation, diagnostic and therapeutic strategies, as well as outcomes of BS in adults, to identify multidisciplinary approach patterns and delineate gaps in contemporary management.

## 2. Materials and Methods

### 2.1. Review Registration

This systematic review was prospectively registered in the Open Science Framework (http://www.osf.io/) with the unique identifying number: 10.17605/OSF.IO/GAM2S. It was conducted and reported in accordance with the Preferred Reporting Items for Systematic Reviews and Meta-Analyses 2020 statement [11]. The PRISMA 2020 Checklist is provided in the Supplementary Materials. 

### 2.2. Search Strategy and Information Sources

A systematic search of the literature was independently performed by two reviewers (E.M., M.F.) using the PubMed and Scopus databases. The search included all records from database inception through 12 June 2026 and used the terms “Boerhaave syndrome” OR “esophageal perforation” to identify studies on the diagnostic and therapeutic management of adult patients with BS. To enhance the sensitivity of the search strategy, the reference lists of all eligible articles were manually examined to identify additional eligible articles.

Following the removal of duplicate studies, titles and abstracts were independently screened by two reviewers (E.M., M.F.). Once non-relevant studies were excluded based on the abstract screening, a full-text review of the remaining studies was performed to confirm their eligibility. Any disagreements that arose between the two reviewers were resolved by a third senior reviewer (D.S.).

### 2.3. Eligibility Criteria and Data Extraction

Studies were considered eligible for inclusion if they satisfied the predefined Patient, Exposure, Comparator, Outcome, and Type of Study (PECOT) framework:

P (*patients*): Adult patients undergoing treatment for BS. Only cases of spontaneous transmural esophageal perforation, without an iatrogenic, traumatic, or other secondary etiology, were considered eligible for inclusion.

E (exposure): Any therapeutic intervention for BS.

C (*comparator*): Not applicable.

O (*outcome*): Clinical characteristics, diagnostic modalities, treatment strategies, and clinical outcomes.

T (*type of study*): Case reports and case series.

Studies were excluded if they were published in a non-English language, did not report data at an individual patient level, included a paediatric population (defined as age < 18 years), or were narrative or prior systematic reviews.

Data were extracted independently from all eligible studies by two reviewers (E.M., M.F.). The following variables were systematically collected: study authorship, year of publication, country of origin, and study design. Patient-level demographic and clinical characteristics, including age, sex, and presenting symptoms, were recorded.

Detailed disease- and treatment-related variables were also extracted, including the anatomical location, laterality, and size of the esophageal perforation, the time interval from symptom onset to diagnosis, the time between hospital admission and initiation of treatment, diagnostic methods utilized, and the type of therapeutic intervention administered. First-line treatment was defined as the initial therapeutic approach implemented following diagnosis, whereas second-line treatment referred to any subsequent intervention required due to failure or complications of the initial management. Additional outcomes of interest included admission to the intensive care unit (ICU), duration of ICU and total hospital stay, postoperative or treatment-related complications, and hospital readmissions. Mortality outcomes (in-hospital and 30-day mortality) and duration of follow-up were also documented.

### 2.4. Methodological Quality, Risk of Bias, and Certainty of Evidence Assessment

Methodological quality and risk of bias of the included studies were independently evaluated by two reviewers (E.M., M.F.) using the Joanna Briggs Institute Critical Appraisal Checklist for Case Reports [12]. Discrepancies arising were resolved by consultation with a third reviewer (D.S.). The certainty of the evidence for the primary outcome, in-hospital mortality, was assessed in accordance with the Grading of Recommendations Assessment, Development and Evaluation (GRADE) framework [13]. The overall quality of evidence was classified as high, moderate, low, or very low after consideration of the following domains: risk of bias, inconsistency of results, indirectness of evidence, imprecision of effect estimates, and potential publication bias.

### 2.5. Statistical Analysis

Continuous variables were reported as mean and standard deviation (SD) or median and interquartile range (IQR). Categorical variables were presented as absolute counts and relative frequencies. Because reporting was incomplete and heterogeneous across the included case reports and case series, analyses were performed using available case data. No imputation of missing values was performed. For each variable, patients with missing or unreported data were excluded only from the analysis of that specific variable. Therefore, denominators vary across variables and are explicitly reported throughout the Results. Potential duplicate reporting was assessed by cross-checking authorship and available patient-level demographic and perforation characteristics across publications; no duplicate patients were identified.

Univariate logistic regression models were constructed with in-hospital mortality, ICU admission, need for second-line treatment, and postoperative complications as dependent variables. For in-hospital mortality, a multivariate logistic regression model was developed in accordance with the rule of thumb, including variables with *p* < 0.2 in the univariate analysis. Backward stepwise selection was applied to obtain the final model. Odds ratios (ORs) with 95% confidence intervals (CIs) are reported. All regression analyses were conducted using complete observations for the variables included in the respective model, without imputation of missing covariate or outcome data. Given the case-based nature of the dataset, heterogeneity in reporting and missingness for several variables, all regression analyses were considered exploratory and hypothesis-generating rather than confirmatory or causal. 

In addition, given the extended publication period of the included literature and the substantial evolution in diagnostic modalities, endoscopic techniques, surgical management and perioperative care over time, a prespecified subgroup analysis restricted to studies published from 2000 onwards was performed. This analysis was intended to assess whether the principal descriptive findings and outcome patterns observed in the overall cohort remained applicable to more contemporary clinical practice. A *p*-value < 0.05 was considered statistically significant. Statistical analysis was performed using R statistical software (version 4.2.2). 

## 3. Results

### 3.1. Study Characteristics and Patient Demographics

**Figure 1:**
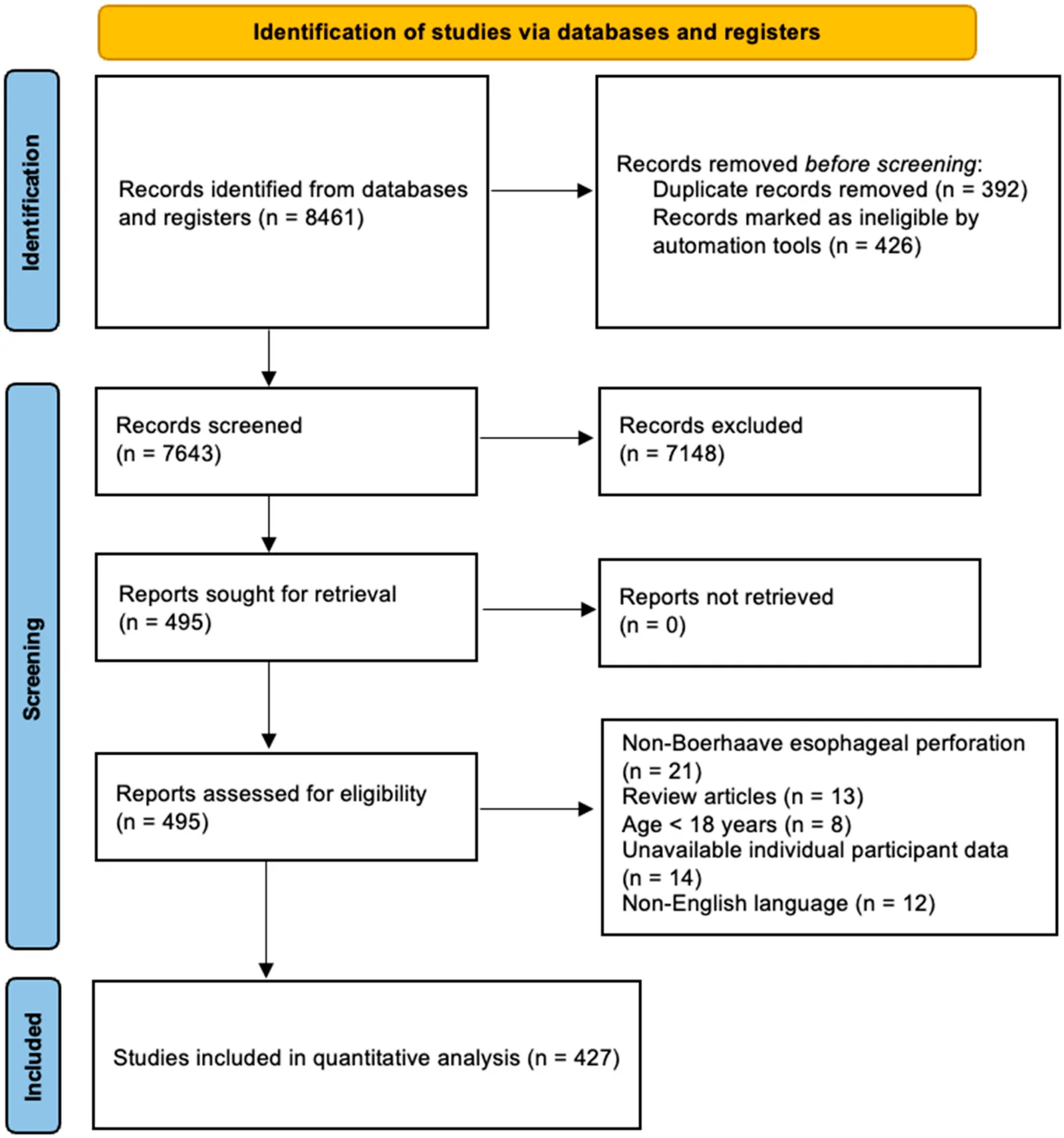
The PRISMA 2020 flowchart of included studies.

A total of 427 studies [14,15,16,17,18,19,20,21,22,23,24,25,26,27,28,29,30,31,32,33,34,35,36,37,38,39,40,41,42,43,44,45,46,47,48,49,50,51,52,53,54,55,56,57,58,59,60,61,62,63,64,65,66,67,68,69,70,71,72,73,74,75,76,77,78,79,80,81,82,83,84,85,86,87,88,89,90,91,92,93,94,95,96,97,98,99,100,101,102,103,104,105,106,107,108,109,110,111,112,113,114,115,116,117,118,119,120,121,122,123,124,125,126,127,128,129,130,131,132,133,134,135,136,137,138,139,140,141,142,143,144,145,146,147,148,149,150,151,152,153,154,155,156,157,158,159,160,161,162,163,164,165,166,167,168,169,170,171,172,173,174,175,176,177,178,179,180,181,182,183,184,185,186,187,188,189,190,191,192,193,194,195,196,197,198,199,200,201,202,203,204,205,206,207,208,209,210,211,212,213,214,215,216,217,218,219,220,221,222,223,224,225,226,227,228,229,230,231,232,233,234,235,236,237,238,239,240,241,242,243,244,245,246,247,248,249,250,251,252,253,254,255,256,257,258,259,260,261,262,263,264,265,266,267,268,269,270,271,272,273,274,275,276,277,278,279,280,281,282,283,284,285,286,287,288,289,290,291,292,293,294,295,296,297,298,299,300,301,302,303,304,305,306,307,308,309,310,311,312,313,314,315,316,317,318,319,320,321,322,323,324,325,326,327,328,329,330,331,332,333,334,335,336,337,338,339,340,341,342,343,344,345,346,347,348,349,350,351,352,353,354,355,356,357,358,359,360,361,362,363,364,365,366,367,368,369,370,371,372,373,374,375,376,377,378,379,380,381,382,383,384,385,386,387,388,389,390,391,392,393,394,395,396,397,398,399,400,401,402,403,404,405,406,407,408,409,410,411,412,413,414,415,416,417,418,419,420,421,422,423,424,425,426,427,428,429,430,431,432,433,434,435,436,437] published between 1961 and 2026 were included (Figure 1), comprising 694 patients diagnosed with BS. The mean age of patients was 57.1 years (SD 16.3). Gender was reported for 649 patients, and the majority were male (80.6%). Precipitating factors were documented in 199 patients. Acute alcohol consumption was identified as the most common trigger (51.8%), either alone or in combination with meal ingestion. Preexisting esophageal pathology was reported in 76 patients, with gastroesophageal reflux disease (40.8%) and Barrett’s esophagus (17.1%) being the most prevalent conditions.

### 3.2. Anatomical Characteristics of Esophageal Perforation

Data on perforation location were available for 473 patients. The distal esophagus was the most frequently impacted segment, accounting for 83.5% of cases, followed by the middle esophagus (11.4%) and upper esophagus (4.0%). Laterality was reported for 235 patients, and perforations most commonly occurred on the left side (77%). Circumferential orientation was available for 74 patients, with posterolateral being the most common (34.7%), followed by posterior (29.2%) and lateral alone (22.2%), while anterior accounted for 8.33% of patients. The median size of the defect was 3 cm (IQR 2 cm). Table 1 summarizes patient and anatomical characteristics.

### 3.3. Clinical Presentation

Data on clinical presentation were available for 570 patients. Vomiting was the most frequently reported presenting symptom (79.5%), followed by chest pain (60.7%), and abdominal pain (35.8%). Respiratory symptoms, including dyspnea (29.0%) and cough (5.6%), were also commonly documented. Systemic manifestations were prevalent, with 67.6% of patients fulfilling the criteria for systemic inflammatory response syndrome (SIRS) at the time of initial diagnosis. The interval between symptom onset and diagnosis was reported for 337 patients. While approximately 45.5% were diagnosed within 24 h, 34.7% of patients experienced diagnostic delays exceeding 72 h.

### 3.4. Diagnostic Evaluation

Diagnostic modalities were reported in 528 patients. Computed tomography (CT) was the most frequent imaging modality (55.5%), followed by endoscopy (22.7%) and water-soluble contrast studies (16.9%). Chest radiography was utilized in 12.5% of cases, primarily as an initial screening tool. In a minority of patients, the diagnosis was established intraoperatively.

### 3.5. Therapeutic Strategies

First-line treatment data were available for 670 patients. Surgical management constituted the predominant therapeutic approach (62.8%), followed by conservative (18.2%) and endoscopic (16.3%) strategies. Among patients undergoing endoscopic therapy, stent placement was the most employed modality (71.5%), followed by clip placement (17.9%) and EVT (8.94%). Surgical interventions varied considerably, with primary repair, either without (60.8%) or with reinforcement (26.1%), being the most frequently performed procedure. Esophagectomy was performed in 17.1%, with reconstruction performed in 8.4% of patients. Lavage and drainage were performed in 58.8%, decortication in 18%, and feeding jejunostomy in 25.8% of patients.

Second-line treatment was required in 40.1% of patients, most frequently including surgical intervention (24.0%) or endoscopic therapy (12.1%). Chest tube placement was performed in 430 patients (87.6%). Among patients with available data (*n* = 201), treatment was initiated within 24 h of hospital admission in 63.2% of cases. Table 2 consolidates the treatment strategy.

### 3.6. Clinical Outcomes

In-hospital mortality was reported in 649 patients and occurred in 18.2% of cases, while 30-day mortality was documented in 644 patients and was 15.4%. ICU admission was reported in 302 patients, of whom 76.5% required critical care. The median length of ICU stay was 10 days (IQR 16), while the median hospital stay was 25 days (IQR 33). 

Post–first-line treatment complications occurred in 300 patients (63.8%), with leakage (17.2%), empyema (11.3%), and sepsis (8.9%) being the most frequently reported adverse events. Stent-related complications included migration in 18.1% of cases, while healing after stent placement was achieved in 75 (79%) out of 95 patients with available follow-up data. Readmissions were documented in 53.7% of reported cases. The median follow-up duration was 6 months (IQR 9). Table 3 summarizes clinical outcomes.

### 3.7. Factors Associated with In-Hospital Mortality

On univariate analysis, increasing age, female sex, delayed diagnosis (>24 h), and the presence of SIRS at initial presentation were significantly associated with increased in-hospital mortality. Increasing age was associated with a higher risk of death, with 5% higher odds of in-hospital death per additional year of age (OR 1.05, 95% CI 1.04–1.07; *p* < 0.001). Female sex was also associated with 1.72-fold higher odds of in-hospital mortality (OR 1.72, 95% CI 1.05–2.77; *p* = 0.029). A diagnostic delay exceeding 24 h was associated with more than a twofold increase in in-hospital mortality risk (OR 2.24, 95% CI 1.23–4.23; *p* = 0.010). Patients presenting with SIRS at initial diagnosis had 5.67 times higher odds of in-hospital mortality (OR 5.67, 95% CI 2.79–13.16; *p* < 0.001).

The multivariable analysis included 199 patients with complete data for age, diagnostic delay, SIRS at diagnosis, and in-hospital mortality, among whom 35 (17.6%) died during hospitalization. In multivariate analysis, age and SIRS at initial diagnosis remained associated with in-hospital mortality. Patients exhibited 5% higher odds of in-hospital mortality for each additional year of age, adjusted for delay to diagnosis and SIRS at initial diagnosis (OR 1.05, 95% CI 1.02–1.08; *p* < 0.001). Similarly, the presence of SIRS was associated with a 9.23-fold increase in the odds of in-hospital mortality adjusted for age and diagnostic delay (OR 9.23, 95% CI 2.58–59.36; *p* = 0.004). In contrast, diagnostic delay did not retain statistical significance after adjustment for confounding factors (Table 4).

### 3.8. Associations Between Treatment Strategy and Clinical Outcomes

In exploratory analysis, conservative management was associated with 88% lower odds for ICU admission (OR 0.12, 95% CI 0.06–0.23; *p* < 0.001) and 54% reduced odds for postoperative complications (OR 0.46, 95% CI 0.27–0.77; *p* = 0.004) compared to surgical management. In addition, conservative treatment was associated with a 39% reduction in the odds of needing second-line therapeutic interventions (OR 0.61, 95% CI 0.37–0.99; *p* = 0.048) compared to surgical management. No statistically significant association was observed between conservative management and in-hospital mortality (OR 1.53, 95% CI 0.92–2.47; *p* = 0.092).

Endoscopic therapy was similarly associated with 81% lower odds of ICU admission compared to surgical management (OR 0.19, 95% CI 0.09–0.41; *p* < 0.001). However, no significant associations were identified between endoscopic treatment and the need for second-line treatment (OR 0.84, 95% CI 0.52–1.35; *p* = 0.49), postoperative complications (OR 1.05, 95% CI 0.61–1.85; *p* = 0.86), and in-hospital mortality (OR 0.50, 95% CI 0.23–0.96; *p* = 0.051). Table 5 summarizes the outcomes.

### 3.9. Subgroup Analysis

A subgroup analysis including studies published after 2000 (333 studies, 509 patients) was conducted to explore potential temporal changes in diagnostic and therapeutic practices (Supplementary Material, Tables S1–S3). The demographic details, anatomical distribution of perforations, and clinical presentation were broadly similar to those observed in the overall cohort. The distal esophagus remained the most frequently affected segment in 83.2% of patients, while vomiting and chest pain continued to represent the most common presenting symptoms in 80.9% and 62.8% of patients. Systemic inflammatory response syndrome was present in 192 patients (64.4%) at the time of diagnosis.

Computed tomography was the predominant diagnostic modality in 70.9% of patients. Surgical management remained the most frequent first-line treatment (59.5%), although endoscopic (21.8%) and conservative approaches (15.5%) were increasingly reported. Despite these developments, treatment strategies remained heterogeneous and clinical outcomes in the post-2000 subgroup remained comparable to those observed in the overall analysis.

### 3.10. Risk of Bias Assessment and Quality of Evidence

The risk of bias assessment for the included studies is presented in the Supplementary Material, Table S4. The certainty of evidence according to the GRADE framework for in-hospital mortality was rated as very low, reflecting the inclusion of case reports, imprecision of effect estimates and the potential for publication bias.

## 4. Discussion

BS has increasingly been managed using non-operative and minimally invasive strategies, reflecting broader shifts toward organ-preserving strategies in esophageal emergencies [5,7,9]. This systematic review and individual patient-level pooled analysis synthesizes data from 427 studies, including 694 adult patients with BS, providing, to our knowledge, the most comprehensive assessment to date of clinical presentation, management strategies, and outcomes. Consistent with the results of previous studies and despite the advent of novel therapeutic options, in-hospital mortality was 18.2%, underlining the severity of BS and the impact of SIRS at initial presentation [438,439].

The identified anatomical and clinical characteristics closely mirror patterns previously described in the literature [6,9]. The distal esophagus was affected more frequently, with a predominance of the left side. This aligns with the recognized structural vulnerability, whereby vomiting-related intraluminal pressurization and mucosal prolapse, combined with asymmetric diaphragmatic traction and reduced structural support, make the left posterolateral wall particularly susceptible to longitudinal full-thickness rupture [6,164]. Vomiting and acute chest pain remained the most frequent presenting symptoms. In addition, the wide range of abdominal and respiratory manifestations highlighted the nonspecific clinical presentation often contributing to diagnostic delays [6,255,440]. The high prevalence of SIRS at initial diagnosis underlines the rapid progression to systemic inflammation once mediastinal contamination occurs [440]. Computed tomography emerged as the predominant diagnostic modality, replacing contrast esophagography and chest x-rays. This shift reflects its superior sensitivity, ranging from 92–100%, in confirming the site of perforation, but also in delineating the extension to adjacent structures, aligning with current guideline recommendations [6,10,440]. However, the present data do not permit comparative assessment of diagnostic accuracy between imaging modalities. Future prospective studies using standardized, risk-stratified diagnostic algorithms are warranted to comparatively evaluate imaging modalities and their diagnostic accuracy across different clinical presentations of BS.

In exploratory multivariable analysis, increasing age and the presence of SIRS at diagnosis remained associated with in-hospital mortality within the available dataset. Although the multivariable analysis included 199 patients with 35 mortality events, the wide confidence interval observed for SIRS reflects substantial uncertainty in the magnitude of this association and warrants cautious interpretation. Given the case-based nature of the underlying evidence, these associations should not be interpreted as validated prognostic predictors and require confirmation in prospective and multicenter cohorts. SIRS should also be interpreted within the historical context of the included literature, as contemporary sepsis assessment has shifted toward Sepsis-3 criteria and organ dysfunction-based severity scores. Its association with mortality therefore reflects the broader physiological derangement and future prospective studies should validate these findings using contemporary severity scores. Although diagnostic delay beyond 24 h has traditionally been perceived as a prognostic indicator [6,7], this effect did not persist after adjustment, suggesting that delay is a surrogate for disease severity rather than an independent driver of mortality. In accordance with the findings of the present study, the systematic review by Vermeulen BD et al. and the multinational retrospective cohort study by Hauge T et al. also demonstrated that delayed intervention, defined as initiation of treatment more than 24 h after symptom onset, was not associated with increased mortality in patients with BS [9,438]. 

The present study also demonstrates the shift in the therapeutic landscape of BS [6,440]. While surgery remained the most common first-line approach, nearly one-third of patients were managed conservatively or endoscopically. In contrast to the multicenter retrospective study by Hauge T et al., which did not demonstrate significant differences in ICU length of stay between treatment strategies, in the present study, conservative and endoscopic approaches were associated with lower rates of ICU admission [9]. However, treatment allocation in BS is intrinsically dependent on clinical presentation, as these strategies are typically reserved for carefully selected patients with contained perforations, hemodynamic stability and absence of sepsis. In contrast, surgery is preferentially employed in more complex cases, including patients with extensive mediastinal contamination, hemodynamic instability, or delayed presentation [6,10]. According to Hauge et al., in-hospital mortality was comparable across the different first-line treatment approaches [9]. The absence of a statistically significant difference in mortality between treatment groups therefore cannot be interpreted as evidence of therapeutic equivalence, but these findings support the recommendations for individualized, organ-preserving management in carefully selected patients, particularly those with hemodynamic stability, contained perforations, and absence of sepsis [6,10].

The high rates of complications and need for second-line interventions across all treatment groups further highlight the complexity of managing BS [7,441]. In the present study, more than 40% of patients required escalation of therapy, reflecting both the severity of BS and the limitations of first-line treatment strategies. While endoscopic therapies have broadened the therapeutic armamentarium, the reported reintervention rates remain high, with 27% of patients requiring endoscopic procedures and 39% of patients undergoing surgical reinterventions [7]. Therefore, their effectiveness is linked to careful patient selection, local expertise, and early diagnosis, while procedure-specific complications, including stent migration, remain a significant challenge [7,10,442].

Among the limitations of this study is the inclusion of case reports and case series, which are inherently subject to selection bias, reporting bias, and incomplete data. The restriction of the search to PubMed/MEDLINE, Scopus and English-language publications may have also introduced selection bias, while inter-rater agreement for study eligibility was not formally quantified, representing an additional methodological limitation. In addition, many included cases were reported over an extended time period, during which diagnostic modalities, surgical techniques, perioperative care, and endoscopic therapies have evolved, potentially confounding outcome assessment and introducing an additional limitation. However, to address this, a subgroup analysis was performed, including studies published after 2000. Despite the comparable outcome patterns compared to the overall cohort, temporal heterogeneity cannot be fully eliminated and should be considered when interpreting the findings. Furthermore, incomplete and heterogeneous reporting across the included studies resulted in missing data and differing denominators, with available-case analysis potentially introducing additional reporting and selection bias. The heterogeneous and incomplete follow-up may have resulted in underestimation of late mortality and complications. In addition, comparisons between conservative, endoscopic, and surgical management are limited by confounding by indication and unequal baseline severity across groups, while important factors including perforation size, extent of contamination, hemodynamic status, comorbidity burden, and timing of intervention were incompletely reported and could not be adequately adjusted for, restricting causal interpretation of outcome differences. Moreover, missing data may have been non-random due to selective reporting inherent to case-based literature, potentially introducing bias into the observed associations despite available-case analysis. In this context, regression analyses were undertaken as exploratory to identify potential patient- and disease-related factors associated with adverse outcomes, which should be interpreted as hypothesis generating requiring validation in prospective studies Despite that, this study represents, to our knowledge, the largest and most comprehensive synthesis of individual patient-level data focusing exclusively on adult patients with BS, allowing detailed analysis of presentation, management, and outcomes, and providing novel insights into prognostic factors.

## 5. Conclusions

BS remains a rare but severe clinical condition associated with significant mortality, primarily driven by systemic inflammation at presentation. Surgery continues to be the most frequently applied treatment, although conservative and endoscopic treatment are increasingly used in carefully selected patients. In exploratory analyses, age and SIRS were associated with mortality, whereas delay to diagnosis did not display independent prognostic significance. Yet, these findings remain hypothesis-generating and require prospective validation. While recent multicenter comparative studies have begun to clarify contemporary management, further prospective collaborative studies focusing on standardized reporting and patient selection are needed to inform treatment algorithms and optimize treatment outcomes.

## Figures and Tables

**Table 1:** Demographic and clinical characteristics.

Number of studies included	427
Study Period	1961–2026
Total patients included	694
Age (mean, SD)	57.1 (16.3)
Gender (N = 649)	
Male	523 (80.6)
Female	126 (19.4)
Perforation site (N = 473)	
Upper	19 (4)
Middle	54 (11.4)
Distal	395 (83.5)
Distal & middle	4 (0.85)
Upper & middle	1 (0.21)
Perforation side (N = 235)	
Left	181 (77)
Right	54 (23)
Perforation’s circumferential orientation (N = 74)	
Anterior	6 (8.33)
Posterior	21 (29.2)
Lateral	16 (22.2)
Posterolateral	25 (34.7)
Anterolateral	2 (2.78)
Bilateral	2 (2.78)
Size of defect (cm, median (IQR))	3 (2)
Symptoms (N = 570)	
Vomiting	453 (79.5)
Hematemesis	64 (11.2)
Chest pain	346 (60.7)
Abdominal pain	204 (35.8)
Back pain	86 (15.1)
Shoulder pain	22 (3.86)
Neck pain	22 (3.86)
Dysphagia	26 (4.56)
Fever	71 (12.5)
Dyspnea	165 (29)
Cough	32 (5.6)
Face and neck swelling	8 (1.4)
Altered mental status	12 (2.11)
SIRS at initial diagnosis (N= 389)	263 (67.6)
Diagnostic modality (N = 528)	
CT	293 (55.5)
Endoscopy	120 (22.7)
Water-soluble contrast study	89 (16.9)
Barium swallow	35 (6.63)
NOS contrast study	94 (17.8)
Chest X-ray	66 (12.5)
Thoracocentesis—chest tube	23 (4.36)
Methylene blue test	21 (3.98)
Intraoperative diagnosis	3 (0.57)
Precipitating factor (N = 199)	
Alcohol consumption	103 (51.8)
Meal ingestion	64 (32.2)
Alcohol & meal ingestion	21 (10.6)
Medication ingestion	7 (3.52)
Bowel preparation for colonoscopy	1 (0.5)
No	3 (1.51)
Preexisting esophageal disease (N = 76)	
GERD	31 (40.8)
Barrett’s esophagus	13 (17.1)
Eosinophilic esophagitis	10 (13.2)
Achalasia	4 (5.3)
Candidiasis	3 (3.9)
Tuberculosis	3 (3.9)
Diverticulum	2 (2.6)
Malignancy	2 (2.6)
Mallory-Weiss	2 (2.6)
History of Boerhaave’s syndrome	7 (9.2)
NOS Dysphagia	8 (10.5)
NOS Esophagitis	4 (5.3)
Time interval from symptom onset to diagnosis (N = 337)	
<12 h	78 (23.2)
12–24 h	75 (22.3)
24–72 h	67 (19.9)
>72 h	117 (34.7)
Time interval from hospital admission to treatment (N = 201)	
<24 h	127 (63.2)
24–72 h	41 (20.4)
72–168 h	19 (9.5)
>1 week	14 (6.9)

**Table 2:** Treatment.

Type of first-line treatment (N = 670)	
Conservative	122 (18.2)
Endoscopic	109 (16.3)
Surgery	421 (62.8)
Endoscopic & surgery	18 (2.69)
Endoscopic treatment (N = 123)	
Stent	88 (71.5)
EVT	11 (8.94)
Clip	22 (17.9)
Stent & clip	2 (1.63)
Surgical approach (N = 301)^1^	
Thoracotomy	183 (60.8)
Thoracoscopy	25 (8.3)
Laparotomy	33 (11)
Laparoscopy	17 (5.6)
Transhiatal	15 (5)
Thoracoscopy & laparoscopy	8 (2.7)
Thoracotomy & laparotomy	17 (5.6)
Thoracotomy & laparoscopy	1 (0.3)
Thoracoscopy & laparotomy	2 (0.7)
Surgery (N = 345) ^1^	
Primary repair	170 (49.3)
Primary repair with reinforcement	90 (26.1)
Esophagectomy with reconstruction	29 (8.4)
Esophagectomy without reconstruction	30 (8.7)
T-tube placement	14 (4.1)
Esophageal diversion	9 (2.6)
Extended gastrectomy	2 (0.6)
Esophageal diversion & extended gastrectomy	1 (0.3)
Decortication	62 (18)
Lavage & drainage	203 (58.8)
Feeding jejunostomy	89 (25.8)
Second-line treatment (N= 479)	
No	287 (59.9)
Conservative	8 (1.7)
Endoscopic	58 (12.1)
Surgery	115 (24)
Yes NOS	11 (2.3)
Chest tube placement (N = 491)	430 (87.6)

^1^ Denominators differ because surgical approach was reported for 301 patients, whereas specific surgical procedure was reported for 345 patients.

**Table 3:** Clinical outcomes.

ICU admissions (N = 302)	231 (76.5)
Length of ICU stay (days, median (IQR))	10 (16)
Length of mechanical ventilation (days, median (IQR))	4 (13)
Length of hospital stay (days, median (IQR))	25 (33)
Post-first-line treatment complications (N = 470)	
No	170 (36.2)
Yes	300 (63.8)
Type of complication (N = 470)	
Leakage	81 (17.2)
Fistula	35 (7.45)
Abscess	21 (4.47)
Stricture	7 (1.49)
Stroke	4 (0.85)
SSI	13 (2.77)
Pleural effusion	28 (6)
Empyema	53 (11.3)
Pneumonia	23(4.89)
Atrial fibrillation	13 (2.77)
Respiratory failure	24 (5.1)
Renal failure	8 (1.7)
Multiorgan failure	18 (3.8)
Sepsis	42 (8.9)
Septic shock	26 (5.5)
Stent migration (N = 105) *	19 (18.1)
Healing after stent (N = 95)	75 (79)
Readmissions (N = 67)	36 (53.7)
In-hospital mortality (N = 649)	118 (18.2)
30-day mortality (N = 644)	99 (15.4)
Follow-up (months, median (IQR))	6 (9)

* Stent migration was assessed among 105 patients who received a stent and had available data.

**Table 4:** Univariate and multivariate analysis for in-hospital mortality.

		Univariate			Multivariate	
	OR	95% CI	*p*-Value **	OR	95% CI	*p*-Value **
**Age** (per 1 year)	1.05	1.04–1.07	**<0.001**	1.05	1.02–1.08	**<0.001**
**Gender**						
Male	—	—				
Female	1.72	1.05–2.77	**0.029**			
**Delay to diagnosis**						
<24 h	—	—		—	—	
>24 h	2.24	1.23–4.23	**0.01**	1.47	0.64–3.51	0.372
**SIRS at diagnosis**						
No *	—	—		—	—	
Yes	5.67	2.79–13.16	**<0.001**	9.23	2.58–59.36	**0.004**

* No SIRS at diagnosis was used as the reference category. ** *p*-values < 0.05 are shown in bold.

**Table 5:** Univariate analysis of clinical outcomes according to treatment strategy.

	Surgery ± Endoscopic Therapy *	Conservative Treatment	Endoscopic Treatment
		OR ** (95% CI), *p*-Value	OR ** (95% CI), *p*-Value
ICU stay	-	0.12 (0.06–0.23), *p* < 0.001	0.19 (0.09–0.41), *p* < 0.001
Second-line treatment	-	0.61 (0.37–0.99), *p* = 0.048	0.84 (0.52–1.35), *p* = 0.49
Postoperative complication	-	0.46 (0.27–0.77), *p* = 0.004	1.05 (0.61–1.85), *p* = 0.86
In-hospital mortality	-	1.53 (0.92–2.47), *p* = 0.092	0.50 (0.23–0.96), *p* = 0.051

* Reference category: surgery (including combined endoscopic + surgery). ** ORs from univariate logistic regression.

## Data Availability

The individual patient-level dataset extracted from the included published reports and used for the present analyses is available from the corresponding author upon reasonable request.

## Supplementary Materials

The following supporting information can be downloaded at: https://www.mdpi.com/article/10.3390/jcm15187032/s1, Table S1: Demographic and clinical characteristics for studies published after 2000; Table S2: Treatment details for studies published after 2000; Table S3: Clinical Outcomes for studies published after 2000; Table S4: Risk of bias assessment using the JBI Critical Appraisal Checklist for Case Reports; PRISMA 2020 Checklist.

## Abbreviations

The following abbreviations are used in this manuscript:

BS	Boerhaave’s syndrome
EVT	Endoscopic vacuum therapy
ICU	Intensive care unit
SD	Standard deviation
IQR	Interquartile range
OR	Odds ratio
SIRS	Systemic inflammatory response syndrome
CT	Computed tomography
