# Supplementary material for: Diagnostic Approach and Therapeutic Management of Spontaneous Esophageal Perforation (Boerhaave’s Syndrome): A Systematic Review and Individual Patient-Level Pooled Analysis
Source: J Clin Med. 2026 Sep 11;15(18):7032. doi: 10.3390/jcm15187032 (PMC13607280; doi:10.3390/jcm15187032)
Supplement: Supplementary file 1 [file jcm-15-07032-s001.zip › jcm-4503339-supplementary Tables.pdf]

Diagnostic Approach and Therapeutic Management of Spontaneous Esophageal Perforation (Boerhaave's Syndrome): A Systematic Review and Individual Patient-Level Pooled Analysis

Evgenia Mela [1,*], Maximos Frountzas [2], Nefeli Tomara [3], Dimitrios Damaskos [4], Nektaria Karangeli [1], Adam Mylonakis [1], Markos Despotidis [1], Aristeidis Sourgiadakis [1], Nikoletta Dimitriou [1], George Tribonias [5] and Dimitrios Schizas [1]

1. First Department of Surgery, National and Kapodistrian University of Athens, Laikon General Hospital, 11527 Athens, Greece; nekkarangeli@gmail.com (N.K.); adam.mylonakis@gmail.com (A.M.); markosd1995@yahoo.gr (M.D.); aristeidis.sou@gmail.com (A.S.); nicole-demetriou@outlook.com (N.D.); schizasad@gmail.com (D.S.)
2. Department of Colorectal Surgery, Royal Marsden Hospital, NHS Foundation Trust, London SW3 6JJ, UK; froumax@hotmail.com
3. Second Propaedeutic Department of Surgery, National and Kapodistrian University of Athens, Laikon General Hospital, 11527 Athens, Greece; neftomara@gmail.com
4. General and Upper GI Surgical Unit, Royal Infirmary of Edinburgh, Edinburgh EH16 4SA, UK; dimitris.damaskos@gmail.com
5. Department of Gastroenterology, Red Cross Hospital, 11526 Athens, Greece; gtrimponias@gmail.com

* Correspondence: evmela@med.uoa.gr

Supplementary Material

Supplementary Tables

Table S1 Demographic and clinical characteristics for studies published after 2000 — *pag. 2*

Table S2 Treatment details for studies published after 2000 — *pag. 4*

Table S3 Clinical Outcomes for studies published after 2000 — *pag. 5*

Table S4 Risk of bias assessment using the JBI Critical Appraisal Checklist for Case Reports — *pag. 6*

Supplementary Tables

Table S1 Demographic and clinical characteristics for studies published after 2000

Number of studies included	333
Study Period	2000-2025
Total patients included	509
Age (years) (mean, SD)	57 (16.9)
Gender (N=472)	
Male	383 (81.1)
Female	89 (18.9)
Perforation site (N=334)	
Upper	14 (4.2)
Middle	37 (11.1)
Distal	278 (83.2)
Distal & middle	4 (1.2)
Upper & middle	1 (0.3)
Perforation side (N=162)	
Left	129 (79.6)
Right	33 (20.4)
Perforation's circumferential orientation (N=46)	
Anterior	3 (6.5)
Posterior	16 (34.8)
Lateral	6 (13)
Posterolateral	18 (39.1)
Anterolateral	2 (4.3)
Bilateral	1 (2.2)
Size of defect (cm, median (IQR))	3 (2)
Symptoms	
Vomiting	351 (80.9)
Hematemesis	43 (9.9)
Chest pain	273(62.8)
Abdominal pain	142 (32.6)
Back pain	57 (13.1)
Shoulder pain	13 (3)
Neck pain	18 (4.1)
Dysphagia	18 (4.1)
Fever	48 (11)
Dyspnea	126 (29)
Cough	27 (6.2)
Face and neck swelling	7 (1.6)
Altered mental status	10 (2.3)
SIRS at initial diagnosis (N= 298)	192 (64.4)
Diagnostic modality	
CT	280 (70.9)
Endoscopy	103 (26)
Water-soluble contrast study	50 (12.6)

Barium swallow	12 (3)
NOS contrast study	63 (15.9)
Chest X-ray	41 (10.4)
Thoracocentesis – Chest tube	11 (2.8)
Methylene blue test	16 (4)
Intraoperative diagnosis	2 (0.5)
Precipitating factor	
Alcohol consumption	81 (55.9)
Meal ingestion	44 (30.3)
Alcohol & meal ingestion	10 (6.9)
Medication ingestion	6 (4.1)
Bowel preparation for colonoscopy	1 (0.7)
No	3 (2.1)
Preexisting esophageal disease	
GERD	27 (37)
Barrett's esophagus	11 (15.1)
Eosinophilic esophagitis	10 (13.7)
Achalasia	4 (5.5)
Candidiasis	3 (4.1)
Tuberculosis	2 (2.7)
Diverticulum	2 (2.7)
Malignancy	2 (2.7)
Mallory-Weiss	2 (2.7)
History of Boerhaave's syndrome	5 (6.8)
NOS Dysphagia	3 (4.1)
NOS Esophagitis	2 (2.7)
Time interval from symptom onset to diagnosis	
< 12 hours	53 (20.9)
12 – 24 hours	52 (20.5)
24 – 72 hours	57 (22.4)
>72 hours	92 (36.2)
Time interval from hospital admission to treatment	
< 24 hours	76 (73.1)
24 – 72 hours	0 (0)
72 – 168 hours	17 (16.3)
> 1 week	11 (10.6)

Values are expressed as n (%), mean (SD), or median (IQR).

Table S2 Treatment details for studies published after 2000

Type first-line treatment	
Conservative	76 (15.5)
Endoscopic	107 (21.8)
Surgery	292 (59.5)
Endoscopic & Surgery	16 (3.3)
Endoscopic treatment	
Stent	86 (71.1)
EVT	11 (9.1)
Clip	22 (18.2)
Stent & Clip	2 (1.7)
Surgical approach	
Thoracotomy	124 (56.1)
Thoracoscopy	23 (10.4)
Laparotomy	21 (9.5)
Laparoscopy	17 (7.7)
Transhiatal	15 (6.8)
Thoracoscopy & Laparoscopy	8 (3.6)
Thoracotomy & Laparotomy	10 (4.5)
Thoracotomy & Laparoscopy	1 (0.5)
Thoracoscopy & Laparotomy	2 (0.9)
Surgery	
Primary repair	113 (47.9)
Primary repair with reinforcement	65 (27.5)
Esophagectomy with reconstruction	17 (7.2)
Esophagectomy without reconstruction	24 (10.2)
T-tube placement	8 (3.4)
Esophageal diversion	7 (3)
Extended gastrectomy	1 (0.4)
Esophageal diversion & extended gastrectomy	1 (0.4)
Decortication	51 (21.6)
Lavage & Drainage	143 (60.6)
Feeding jejunostomy	64 (27.1)
Second-line treatment	
No	216 (62.1)
Conservative	3 (0.9)
Endoscopic	53 (15.2)
Surgery	69 (14.8)
Yes NOS	11 (3.2)
Chest tube placement (N=369)	322 (87.3)

Values are expressed as n (%).

Table S3 Clinical Outcomes for studies published after 2000

ICU admissions (N=249)	197 (79.1)
Length of ICU stay (days, median(IQR))	10 (16)
Length of mechanical ventilation (days, median(IQR))	3 (5.5)
Length of hospital stay (days, median(IQR))	27 (33)
Post-first line treatment complications	
No	329 (64.6)
Yes	180 (35.4)
Type of complication (N=330)	
Leakage	63 (19.1)
Fistula	22 (6.7)
Abscess	15 (4.5)
Stricture	5 (1.5)
Stroke	3 (0.9)
SSI	9 (2.7)
Pleural effusion	20 (6.1)
Empyema	29 (8.8)
Pneumonia	16 (4.8)
Atrial fibrillation	11 (3.3)
Respiratory failure	18 (5.5)
Renal failure	3 (0.9)
Multiorgan failure	16 (4.8)
Sepsis	29 (8.8)
Septic shock	14 (4.2)
Stent migration (N=103)	19 (18.4)
Healing after stent (N=94)	74 (78.7)
Readmissions (N=58)	3 (5.2)
In-hospital mortality (N=479)	77 (16.1)
30-day mortality (N=472)	66 (14)
Follow-up (months, median (IQR))	6 (9)

Values are expressed as n (%) or median (IQR).

Table S4 Risk of bias assessment using the JBI Critical Appraisal Checklist for Case Reports

**Author**	**1**	**2**	**3**	**4**	**5**	**6**	**7**	**8**	**Overall appraisal**
Vest MT et al. [14]	Yes	Yes	Yes	Yes	Yes	Yes	Unclear	Yes	Include
Pinto MJF et al. [15]	Yes	Unclear	Yes	Yes	Yes	Yes	Yes	Yes	Include
He X et al. [16]	Yes	Yes	Yes	Yes	Yes	Yes	Yes	Yes	Include
Pasternak A et al. [17]	Yes	Yes	Yes	Yes	Yes	Yes	Yes	Yes	Include
Chen A et al. [18]	Yes	Yes	Yes	Yes	Yes	Yes	Yes	Yes	Include
Ahmad W et al. [19]	Yes	Unclear	Yes	Yes	Yes	Yes	Not applicable	Yes	Include
Vaidya S et al. [20]	Yes	Yes	Yes	Yes	Yes	Yes	Yes	Yes	Include
Cameron JL et al. [21]	Yes	Yes	Yes	Yes	Yes	Yes	Unclear	Yes	Include
Szeliga J et al. [22]	Yes	Yes	Yes	Yes	Yes	Yes	Yes	Yes	Include
Ferreiro-Iglesias R et al. [23]	Yes	Unclear	Yes	Yes	Yes	Yes	Not applicable	Yes	Include
Lieu MT et al. [24]	Yes	Yes	Yes	Yes	Yes	Yes	Yes	Yes	Include
Chalikonda D. et al. [25]	Yes	Yes	Yes	Yes	Yes	Yes	Yes	Yes	Include
Dinic BR et al. [26]	Yes	Yes	Yes	Yes	Yes	Yes	Yes	Yes	Include
Al-Zahir AA et al. [27]	Yes	Yes	Yes	Yes	Yes	Yes	Not applicable	Yes	Include
Yap D et al. [28]	Yes	Yes	Yes	Yes	Yes	Yes	Yes	Yes	Include
Olivero R. [29]	Yes	Yes	Yes	Yes	Yes	Yes	Yes	Yes	Include
Bayram AS et al. [30]	Yes	Unclear	Yes	Yes	Yes	Yes	Yes	Yes	Include
Swol J et al. [31]	Yes	Yes	Yes	Yes	Yes	Not applicable	Not applicable	Yes	Include

Ekladious A et al. [32]	Yes	Yes	Yes	Yes	Yes	Yes	Yes	Yes	Include
Ali D et al. [33]	Yes	Yes	Yes	Yes	Yes	Yes	Not applicable	Yes	Include
Kim YE et al. [34]	Yes	Yes	Yes	Yes	Yes	Yes	Yes	Yes	Include
Atie M et al. [35]	Yes	Yes	Yes	Yes	Yes	Yes	Not applicable	Yes	Include
Teblick A et al. [36]	Yes	Yes	Yes	Yes	Yes	Yes	Yes	Yes	Include
Emmanouilidis N et al. [37]	Yes	Yes	Yes	Yes	Yes	Yes	Yes	Yes	Include
Cucci M et al. [38]	Yes	Yes	Yes	Yes	Not applicable	Not applicable	Not applicable	Yes	Include
Kopelman Y et al. [39]	Yes	Yes	Yes	Yes	Yes	Yes	Not applicable	Yes	Include
Lucendo AJ et al. [40]	Yes	Yes	Yes	Yes	Yes	Yes	Yes	Yes	Include
Shemesh A et al. [41]	Yes	Unclear	Yes	Yes	Yes	Yes	Yes	Yes	Include
Aref H et al. [42]	Yes	Yes	Yes	Yes	Yes	Yes	Yes	Yes	Include
Okamoto H et al. [43]	Yes	Yes	Yes	Yes	Yes	Yes	Yes	Yes	Include
Anwar J et al. [44]	Yes	Yes	Yes	Yes	Yes	Yes	Unclear	Yes	Include
Carrozza F et al. [45]	Yes	Yes	Yes	Yes	Yes	Yes	Unclear	Yes	Include
Chae RP et al. [46]	Yes	Yes	Yes	Yes	Yes	Yes	Yes	Yes	Include
Vallabhajosyula S et al. [47]	Yes	Yes	Yes	Yes	Yes	Yes	Yes	Yes	Include
Ibrahim-zada I et al. [48]	Yes	Yes	Yes	Yes	Yes	Yes	Yes	Yes	Include
James TW et al. [49]	Yes	Yes	Yes	Yes	Yes	Yes	Not applicable	Yes	Include
Kanzaki R et al. [50]	Yes	Yes	Yes	Yes	Yes	Yes	Not applicable	Yes	Include
Tzeng C et al. [51]	Yes	Yes	Yes	Yes	Yes	Yes	Not applicable	Yes	Include
Barakat MT et al. [52]	Yes	Yes	Yes	Yes	Yes	Yes	Yes	Yes	Include
Kincaid K et al. [53]	Yes	Yes	Yes	Yes	Yes	Yes	Not applicable	Yes	Include
Ishikawa Y et al. [54]	Yes	Unclear	Yes	Yes	Yes	Yes	Yes	Yes	Include
Ijaz M et al. [55]	Yes	Yes	Yes	Yes	Yes	Not applicable	Not applicable	Yes	Include

Tonolini M et al. [56]	Yes	Yes	Yes	Yes	Yes	Yes	Unclear	Yes	Include
Bona D et al. [57]	Yes	Yes	Yes	Yes	Yes	Yes	Yes	Yes	Include
Gupta RK et al. [58]	Yes	Yes	Yes	Yes	Yes	Yes	Not applicable	Yes	Include
De Groote K et al. [59]	Yes	Yes	Yes	Yes	Yes	Yes	Yes	Yes	Include
Yagnik VD [60]	Yes	Yes	Yes	Yes	Yes	Yes	Yes	Yes	Include
Suzuki M et al. [61]	Yes	Yes	Yes	Yes	Yes	Yes	Yes	Yes	Include
Paily A et al. [62]	Yes	Yes	Yes	Yes	Yes	Yes	Not applicable	Yes	Include
Barnett D et al. [63]	Yes	Yes	Yes	Yes	Yes	Yes	Yes	Yes	Include
Davies J et al. [64]	Yes	Yes	Yes	Yes	Not applicable	Not applicable	Not applicable	Yes	Include
Anwuzia-Iwegbu C et al. [65]	Yes	Yes	Yes	Yes	Yes	Yes	Not applicable	Yes	Include
Musala C et al. [66]	Yes	Yes	Yes	Yes	Yes	Yes	Not applicable	Yes	Include
Dichtl K et al. [67]	Yes	Yes	Yes	Yes	Yes	Yes	Yes	Yes	Include
Lee W et al. [68]	Yes	Yes	Yes	Yes	Yes	Yes	Not applicable	Yes	Include
Tamatey MN et al. [69]	Yes	Yes	Yes	Yes	Yes	Yes	Yes	Yes	Include
Kimura-Kataoka K et al. [70]	Yes	Yes	Yes	Yes	Not applicable	Not applicable	Not applicable	Yes	Include
Oh MK et al. [71]	Yes	Yes	Yes	Yes	Yes	Yes	Not applicable	Yes	Include
Ramhamadany E et al. [72]	Yes	Yes	Yes	Yes	Yes	Yes	Not applicable	Yes	Include
Lakshmanadoss U et al. [73]	Yes	Yes	Yes	Yes	Yes	Yes	Not applicable	Yes	Include
Czopnik P et al. [74]	Yes	Yes	Yes	Yes	Yes	Yes	Yes	Yes	Include
González-Haba M et al. [75]	Yes	Yes	Yes	Yes	Yes	Yes	Yes	Yes	Include
Shen G et al. [76]	Yes	Yes	Yes	Yes	Yes	Yes	Yes	Yes	Include
Kobara H et al. [77]	Yes	Yes	Yes	Yes	Yes	Yes	Not applicable	Yes	Include
Ganguly A et al. [78]	Yes	Yes	Yes	Yes	Yes	Yes	Yes	Yes	Include

Vinnamala S et al. [79]	Yes	Yes	Yes	Yes	Yes	Yes	Not applicable	Yes	Include
Van Weyenberg SJ et al.[80]	Yes	Yes	Yes	Yes	Yes	Yes	Not applicable	Yes	Include
Salvador Baudet J et al. [81]	Yes	Yes	Yes	Yes	Yes	Yes	Unclear	Yes	Include
O'Kelly F et al. [82]	Yes	Yes	Yes	Yes	Yes	Yes	Not applicable	Yes	Include
Kimberley KL et al. [83]	Yes	Yes	Yes	Yes	Yes	Yes	Yes	Yes	Include
Zimmer V. [84]	Yes	Yes	Yes	Yes	Yes	Yes	Unclear	Yes	Include
Kundra M et al. [85]	Yes	Yes	Yes	Yes	Yes	Yes	Not applicable	Yes	Include
Larrieu AJ et al. [86]	Yes	Yes	Yes	Yes	Yes	Yes	Yes	Yes	Include
Loske G et al. [87]	Yes	Yes	Yes	Yes	Yes	Yes	Yes	Yes	Include
Naitoh H et al. [88]	Yes	Yes	Yes	Yes	Yes	Yes	Yes	Yes	Include
Ng CS et al. [89]	Yes	Yes	Yes	Yes	Yes	Yes	Yes	Yes	Include
Keane M et al. [90]	Yes	Yes	Yes	Yes	Yes	Yes	Yes	Yes	Include
Barbara DW et al. [91]	Yes	Yes	Yes	Yes	Yes	Yes	Not applicable	Yes	Include
Henderson JA et al. [92]	Yes	Yes	Yes	Yes	Yes	Yes	Yes	Yes	Include
Korczynski P et al. [93]	Yes	Yes	Yes	Yes	Yes	Yes	Yes	Yes	Include
Neff C et al. [94]	Yes	Yes	Yes	Yes	Yes	Yes	Yes	Yes	Include
Ivey TD et al. [95]	Yes	Yes	Yes	Yes	Yes	Yes	Not applicable	Yes	Include
Blencowe NS et al. [96]	Yes	Yes	Yes	Yes	Yes	Unclear	Unclear	Yes	Include
Ho AFW et al. [97]	Yes	Yes	Yes	Yes	Yes	Unclear	Unclear	Yes	Include
Fernandes SR et al. [98]	Yes	Yes	Yes	Yes	Yes	Yes	Not applicable	Yes	Include
Paluszkiewicz P et al. [99]	Yes	Yes	Yes	Yes	Yes	Yes	Not applicable	Yes	Include
Matsuda A et al. [100]	Yes	Yes	Yes	Yes	Yes	Yes	Not applicable	Yes	Include
Thapa AS et al. [101]	Yes	Yes	Yes	Yes	Yes	Yes	Yes	Yes	Include
Dżeljilji A et al. [102]	Yes	Yes	Yes	Yes	Yes	Yes	Yes	Yes	Include
Shiber JR et al. [103]	Yes	Yes	Yes	Yes	Yes	Not applicable	Not applicable	Yes	Include

Bruno MS et al. [104]	Yes	Yes	Yes	Yes	Yes	Not applicable	Not applicable	Yes	Include
Clément R et al. [105]	Yes	Unclear	Yes	Yes	Yes	Not applicable	Not applicable	Yes	Include
Grau Pérez M et al. [106]	Yes	Yes	Yes	Yes	Yes	Yes	Not applicable	Yes	Include
Wilson DR et al. [107]	Yes	Yes	Yes	Yes	Yes	Yes	Not applicable	Yes	Include
Sato H et al. [108]	Yes	Yes	Yes	Yes	Yes	Yes	Yes	Yes	Include
Nitta J et al. [109]	Yes	Yes	Yes	Yes	Yes	Yes	Yes	Yes	Include
Gurvits GE. [110]	Yes	Yes	Yes	Yes	Yes	Yes	Unclear	Yes	Include
Callaghan J. [111]	Yes	Yes	Yes	Yes	Yes	Yes	Yes	Yes	Include
Han NJ et al. [112]	Yes	Yes	Yes	Yes	Yes	Yes	Not applicable	Yes	Include
Kim JH et al. [113]	Yes	Yes	Yes	Yes	Yes	Yes	Not applicable	Yes	Include
Ng P. [114]	Yes	Yes	Yes	Yes	Yes	Yes	Yes	Yes	Include
Arens A et al. [115]	Yes	Yes	Yes	Yes	Yes	Yes	Yes	Yes	Include
Do YW et al. [116]	Yes	Yes	Yes	Yes	Yes	Yes	Unclear	Yes	Include
Li A et al. [117]	Yes	Yes	Yes	Yes	Yes	Yes	Unclear	Yes	Include
Rodriguez-Infante A et al. [118]	Yes	Yes	Yes	Yes	Yes	Yes	Yes	Yes	Include
Johnson HM et al. [119]	Yes	Yes	Yes	Yes	Yes	Yes	Yes	Yes	Include
Cayci HM et al. [120]	Yes	Yes	Yes	Yes	Yes	Yes	Not applicable	Yes	Include
delCastillo J et al. [121]	Yes	Yes	Yes	Yes	Yes	Yes	Yes	Yes	Include
Humphreys ML et al. [122]	Yes	Yes	Yes	Yes	Yes	Yes	Unclear	Yes	Include
Chapman TP et al. [123]	Yes	Yes	Yes	Yes	Yes	Yes	Yes	Yes	Include
Beason HF et al. [124]									
Ngo E et al. [125]	Yes	Yes	Yes	Yes	Yes	Yes	Yes	Yes	Include
Ashrafian H et al. [126]	Yes	Yes	Yes	Yes	Yes	Yes	Yes	Yes	Include
Wu G et al. [127]	Yes	Yes	Yes	Yes	Yes	Yes	Yes	Yes	Include

Ghassemi KF et al. [128]	Yes	Yes	Yes	Yes	Yes	Yes	Yes	Yes	Include
Nensa F et al. [129]	Yes	Yes	Yes	Yes	Yes	Yes	Unclear	Yes	Include
Broadbent K et al. [130]	Yes	Yes	Yes	Yes	Yes	Yes	Not applicable	Yes	Include
Santini M et al. [131]	Yes	Yes	Yes	Yes	Yes	Yes	Yes	Yes	Include
Yagnik VD et al. [132]	Yes	Yes	Yes	Yes	Yes	Yes	Not applicable	Yes	Include
White S. [133]	Yes	Yes	Yes	Yes	Yes	Yes	Not applicable	Yes	Include
Adam A et al. [134]	Yes	Yes	Yes	Yes	Yes	Yes	Yes	Yes	Include
Tanaka R et al. [135]	Yes	Yes	Yes	Yes	Yes	Yes	Yes	Yes	Include
Ota K et al. [136]	Yes	Yes	Yes	Yes	Yes	Yes	Yes	Yes	Include
Rokszin R et al. [137]	Yes	Yes	Yes	Yes	Yes	Yes	Yes	Yes	Include
Schneider SM et al. [138]	Yes	Yes	Yes	Yes	Yes	Yes	Unclear	Yes	Include
Anderson RE et al. [139]	Yes	Yes	Yes	Yes	Yes	Yes	Yes	Yes	Include
Ruttan TK et al. [140]	Yes	Yes	Yes	Yes	Yes	Yes	Unclear	Yes	Include
Scharl M et al. [141]	Yes	Yes	Yes	Yes	Yes	Yes	Unclear	Yes	Include
Klein PE et al. [142]	Yes	Yes	Yes	Yes	Yes	Yes	Yes	Yes	Include
Ali N et al. [143]	Yes	Yes	Yes	Yes	Yes	Yes	Yes	Yes	Include
McLean MM et al. [144]	Yes	Yes	Yes	Yes	Yes	Unclear	Unclear	Yes	Include
Reeder LB et al. [145]	Yes	Yes	Yes	Yes	Yes	Yes	Yes	Yes	Include
Lujan HJ et al. [146]	Yes	Yes	Yes	Yes	Yes	Yes	Yes	Yes	Include
Khan OA et al. [147]	Yes	Yes	Yes	Yes	Yes	Yes	Not applicable	Yes	Include
Ieta K et al. [148]	Yes	Yes	Yes	Yes	Yes	Yes	Yes	Yes	Include
Maurya VK et al. [149]	Yes	Yes	Yes	Yes	Yes	Yes	Not applicable	Yes	Include
Ashok K et al. [150]	Yes	Yes	Yes	Yes	Yes	Yes	Not applicable	Yes	Include
Catarino Santos S et al. [151]	Yes	Yes	Yes	Yes	Yes	Yes	Yes	Yes	Include

Valdivielso-Cortáza E et al. [152]	Yes	Yes	Yes	Yes	Yes	Yes	Not applicable	Yes	Include
Janjua KJ. [153]	Yes	Yes	Yes	Yes	Yes	Yes	Yes	Yes	Include
Saito S et al. [154]	Yes	Yes	Yes	Yes	Yes	Yes	Not applicable	Yes	Include
Ashrafi AS et al. [155]	Yes	Yes	Yes	Yes	Yes	Yes	Yes	Yes	Include
Singh GS et al. [156]	Yes	Yes	Yes	Yes	Yes	Yes	Unclear	Yes	Include
Smith JS et al. [157]	Yes	Yes	Yes	Yes	Yes	Unclear	Unclear	Yes	Include
Bhargava J et al. [158]	Yes	Yes	Yes	Yes	Yes	Yes	Unclear	Yes	Include
Nguyen Ho L et al. [159]	Yes	Yes	Yes	Yes	Yes	Yes	Not applicable	Yes	Include
Hill AG et al. [160]	Yes	Yes	Yes	Yes	Yes	Yes	Yes	Yes	Include
Jagminas L et al. [161]	Yes	Yes	Yes	Yes	Yes	Yes	Yes	Yes	Include
Craven GG et al. [162]	Yes	Yes	Yes	Yes	Yes	Yes	Yes	Yes	Include
Reardon ES et al. [163]	Yes	Yes	Yes	Yes	Yes	Yes	Not applicable	Yes	Include
Kuwano H et al. [164]	Yes	Yes	Yes	Yes	Yes	Yes	Yes	Yes	Include
Tanaka H et al. [165]	Yes	Yes	Yes	Yes	Yes	Yes	Not applicable	Yes	Include
Ould-Nana I et al. [166]	Yes	Yes	Yes	Yes	Yes	Yes	Unclear	Yes	Include
Spapen J et al. [167]	Yes	Yes	Yes	Yes	Yes	Yes	Yes	Yes	Include
Sohoni CA. [168]	Yes	Yes	Yes	Yes	Yes	Yes	Yes	Yes	Include
Adhikari DR et al. [169]	Yes	Yes	Yes	Yes	Yes	Yes	Unclear	Yes	Include
Truyens M et al. [170]	Yes	Yes	Yes	Yes	Yes	Yes	Yes	Yes	Include
van der Weg G et al. [171]	Yes	Yes	Yes	Yes	Yes	Yes	Yes	Yes	Include
Grobmyer SR et al. [172]	Yes	Yes	Yes	Yes	Yes	Yes	Unclear	Yes	Include
Yu JY et al. [173]	Yes	Yes	Yes	Yes	Yes	Yes	Not applicable	Yes	Include
Nakano T et al. [174]	Yes	Yes	Yes	Yes	Yes	Yes	Not applicable	Yes	Include
Toelen C et al. [175]	Yes	Yes	Yes	Yes	Yes	Yes	Yes	Yes	Include

Alkhuja S et al. [176]	Yes	Yes	Yes	Yes	Yes	Yes	Unclear	Yes	Include
Chung MG et al. [177]	Yes	Yes	Yes	Yes	Yes	Yes	Yes	Yes	Include
Rassameehiran S et al. [178]	Yes	Yes	Yes	Yes	Yes	Yes	Yes	Yes	Include
Venø S et al. [179]	Yes	Yes	Yes	Yes	Yes	Yes	Yes	Yes	Include
Jackson WE et al. [180]	Yes	Yes	Yes	Yes	Yes	Yes	Unclear	Yes	Include
Haba Y et al. [181]	Yes	Yes	Yes	Yes	Yes	Yes	Yes	Yes	Include
Veltri A et al. [182]	Yes	Yes	Yes	Yes	Yes	Yes	Yes	Yes	Include
Curci JJ et al. [183]	Yes	Yes	Yes	Yes	Yes	Yes	Yes	Yes	Include
Malik UF et al. [184]	Yes	Yes	Yes	Yes	Yes	Yes	Yes	Yes	Include
Lewis AM et al. [185]	Yes	Yes	Yes	Yes	Yes	Yes	Unclear	Yes	Include
Jougon J et al. [186]	Yes	Yes	Yes	Yes	Yes	Yes	Yes	Yes	Include
Hingston CD et al. [187]	Yes	Yes	Yes	Yes	Yes	Yes	Unclear	Yes	Include
Craik JD et al. [188]	Yes	Yes	Yes	Yes	Yes	Yes	Unclear	Yes	Include
Ayeni JP et al. [189]	Yes	Yes	Yes	Yes	Yes	Yes	Unclear	Yes	Include
Onyeka WO et al. [190]	Yes	Yes	Yes	Yes	Yes	Yes	Yes	Yes	Include
Kollmar O et al. [191]	Yes	Yes	Yes	Yes	Yes	Yes	Yes	Yes	Include
Corns RA et al. [192]	Yes	Yes	Yes	Yes	Yes	Yes	Unclear	Yes	Include
Darrien JH et al. [193]	Yes	Yes	Yes	Yes	Yes	Yes	Yes	Yes	Include
Spinoglio G et al. [194]	Yes	Yes	Yes	Yes	Yes	Yes	Unclear	Yes	Include
Ghatak T et al. [195]	Yes	Yes	Yes	Yes	Yes	Yes	Yes	Yes	Include
Ashrafi AS et al. [196]	Yes	Yes	Yes	Yes	Yes	Yes	Not applicable	Yes	Include
McVay CB et al. [197]	Yes	Yes	Yes	Yes	Yes	Yes	Yes	Yes	Include
Schwartz JA et al. [198]	Yes	Yes	Yes	Yes	Yes	Yes	Yes	Yes	Include
Spronk PE et al. [199]	Yes	Yes	Yes	Yes	Yes	Yes	Yes	Yes	Include

Patel MS et al. [200]	Yes	Yes	Yes	Yes	Yes	Yes	Yes	Yes	Include
Prichard R et al. [201]	Yes	Yes	Yes	Yes	Yes	Yes	Yes	Yes	Include
Atallah FN et al. [202]	Yes	Yes	Yes	Yes	Yes	Yes	Not applicable	Yes	Include
Bailey C et al. [203]	Yes	Yes	Yes	Yes	Yes	Yes	Not applicable	Yes	Include
Joyce M et al. [204]	Yes	Yes	Yes	Yes	Yes	Yes	Not applicable	Yes	Include
Landen S et al. [205]	Yes	Yes	Yes	Yes	Yes	Yes	Yes	Yes	Include
Drury M et al. [206]	Yes	Yes	Yes	Yes	Yes	Yes	Yes	Yes	Include
Babic T et al. [207]	Yes	Yes	Yes	Yes	Yes	Yes	Not applicable	Yes	Include
Zenone EA et al. [208]	Yes	Yes	Yes	Yes	Yes	Yes	Yes	Yes	Include
Matsushita M et al. [209]	Yes	Yes	Yes	Yes	Yes	Yes	Not applicable	Yes	Include
Curtis DL et al. [210]	Yes	Yes	Yes	Yes	Yes	Yes	Yes	Yes	Include
Witz M et al. [211]	Yes	Yes	Yes	Yes	Yes	Yes	Yes	Yes	Include
Troum S et al. [212]	Yes	Yes	Yes	Yes	Yes	Yes	Not applicable	Yes	Include
Mikami R et al. [213]	Yes	Yes	Yes	Yes	Yes	Yes	Unclear	Yes	Include
Jenkins IR et al. [214]	Yes	Yes	Yes	Yes	Yes	Yes	Yes	Yes	Include
Sabanathan S et al. [215]	Yes	Yes	Yes	Yes	Yes	Yes	Yes	Yes	Include
Sekiya S et al. [216]	Yes	Yes	Yes	Yes	Yes	Yes	Yes	Yes	Include
Tsalis K et al. [217]	Yes	Yes	Yes	Yes	Yes	Yes	Yes	Yes	Include
Hutzelmann A et al. [218]	Yes	Yes	Yes	Yes	No	No	No	Yes	Include
Türüt H et al. [219]	Yes	Yes	Yes	Yes	Yes	Yes	Not applicable	Yes	Include
Maglinte DD et al. [220]	Yes	Yes	Yes	Yes	Yes	Yes	Yes	Yes	Include
Kyriacou DN. [221]	Yes	Yes	Yes	Yes	Yes	Yes	Yes	Yes	Include
Hashmi MAR et al. [222]	Yes	Yes	Yes	Yes	Yes	Yes	Yes	Yes	Include
Foroulis CN et al. [223]	Yes	Yes	Yes	Yes	Yes	Yes	Yes	Yes	Include

Nabili S et al. [224]	Yes	Yes	Yes	Yes	Yes	Yes	Not applicable	Yes	Include
Davies AP et al. [225]	Yes	Yes	Yes	Yes	Yes	Yes	Not applicable	Yes	Include
Munemura T et al. [226]	Yes	Yes	Yes	Yes	Yes	Yes	Yes	Yes	Include
Harries K et al. [227]	Yes	Yes	Yes	Yes	Yes	Yes	Not applicable	Yes	Include
Kallis P et al. [228]	Yes	Yes	Yes	Yes	Yes	Yes	Not applicable	Yes	Include
Harikrishnan S et al. [229]	Yes	Yes	Yes	Yes	Yes	Yes	Unclear	Yes	Include
Wei L et al. [230]	Yes	Yes	Yes	Yes	Yes	Yes	Unclear	Yes	Include
Levy F et al. [231]	Yes	Yes	Yes	Yes	Yes	Yes	Yes	Yes	Include
Glenny RW et al. [232]	Yes	Yes	Yes	Yes	Yes	Yes	Yes	Yes	Include
Attia R et al. [233]	Yes	Yes	Yes	Yes	Yes	Yes	Unclear	Yes	Include
Loske G et al. [234]	Yes	Yes	Yes	Yes	Yes	Yes	Not applicable	Yes	Include
Trueman D. [235]	Yes	Yes	Yes	Yes	Yes	Yes	Yes	Yes	Include
Gopalan R et al. [236]	Yes	Yes	Yes	Yes	Yes	Yes	Unclear	Yes	Include
Smyth AR et al. [237]	Yes	Yes	Yes	Yes	Yes	Yes	Yes	Yes	Include
Achem SR. [238]	Yes	Yes	Yes	Yes	Yes	Unclear	Unclear	Yes	Include
Chan-Tack KM. [239]	Yes	Yes	Yes	Yes	Yes	Yes	Not applicable	Yes	Include
Weil RJ. [240]	Yes	Yes	Yes	Yes	Yes	Yes	Yes	Yes	Include
Klausner JM et al. [241]	Yes	Yes	Yes	Yes	Yes	Yes	Yes	Yes	Include
Dominguez A et al. [242]	Yes	Yes	Yes	Yes	Yes	Yes	Not applicable	Yes	Include
Mota HJ et al. [243]	Yes	Yes	Yes	Yes	Yes	Yes	Yes	Yes	Include
Ho LN et al. [244]	Yes	Yes	Yes	Yes	Yes	Yes	Yes	Yes	Include
Baird J et al. [245]	Yes	Yes	Yes	Yes	Yes	Yes	Unclear	Yes	Include
Achem SR. [246]	Yes	Yes	Yes	Yes	Yes	Unclear	Unclear	Yes	Include
Lee TC et al. [247]	Yes	Yes	Yes	Yes	Yes	Unclear	Unclear	Yes	Include
Petruzziello L et al. [248]	Yes	Yes	Yes	Yes	Yes	Yes	Yes	Yes	Include

Franceschi F et al. [249]	Yes	Yes	Yes	Yes	Yes	Yes	Yes	Yes	Include
Bolooki H et al. [250]	Yes	Yes	Yes	Yes	Yes	Yes	Yes	Yes	Include
Kochar T et al. [251]	Yes	Yes	Yes	Yes	Yes	Yes	Yes	Yes	Include
Rochford M et al. [252]	Yes	Yes	Yes	Yes	Yes	Unclear	Not applicable	Yes	Include
Ampollini L et al. [253]	Yes	Yes	Yes	Yes	Yes	Yes	Yes	Yes	Include
Cohen MS et al. [254]	Yes	Yes	Yes	Yes	Yes	Yes	Yes	Yes	Include
Ribeiro TA et al. [255]	Yes	Yes	Yes	Yes	Yes	Yes	Unclear	Yes	Include
Eubanks PJ et al. [256]	Yes	Yes	Yes	Yes	Yes	Yes	Yes	Yes	Include
Scott HJ et al. [257]	Yes	Yes	Yes	Yes	Yes	Yes	Yes	Yes	Include
Aloreidi K et al. [258]	Yes	Yes	Yes	Yes	Yes	Yes	Yes	Yes	Include
Dumonceau JM et al. [259]	Yes	Yes	Yes	Yes	Yes	Yes	Not applicable	Yes	Include
Liu L et al. [260]	Yes	Yes	Yes	Yes	Yes	Yes	Not applicable	Yes	Include
Mwamure P et al. [261]	Yes	Yes	Yes	Yes	Yes	Yes	Unclear	Yes	Include
Dieckhaus KD et al. [262]	Yes	Yes	Yes	Yes	Yes	Unclear	Unclear	Yes	Include
Nehra D et al. [263]	Yes	Yes	Yes	Yes	Yes	Yes	Yes	Yes	Include
D'Journo XB et al. [264]	Yes	Yes	Yes	Yes	Yes	Yes	Yes	Yes	Include
Gubbins GP et al. [265]	Yes	Yes	Yes	Yes	Yes	Yes	Yes	Yes	Include
Teh E et al. [266]	Unclear	Yes	Yes	Yes	Yes	Yes	Yes	Yes	Include
Vyas D. [267]	Yes	Yes	Yes	Yes	Yes	Yes	Unclear	Yes	Include
Reddy S et al. [268]	Yes	Yes	Yes	Yes	Yes	Yes	Yes	Yes	Include
Reñé M et al. [269]	Yes	Yes	Yes	Yes	Yes	Yes	Yes	Yes	Include
Tariq T et al. [270]	Yes	Yes	Yes	Yes	Yes	Yes	Not applicable	Yes	Include
Razi E et al. [271]	Yes	Yes	Yes	Yes	Yes	Yes	Yes	Yes	Include
Baric A. [272]	Yes	Yes	Yes	Yes	Yes	Yes	Yes	Yes	Include
Uehara DT et al. [273]	Yes	Yes	Yes	Yes	Yes	Yes	Yes	Yes	Include

Modi BP et al. [274]	Yes	Yes	Yes	Yes	Yes	Yes	Not applicable	Yes	Include
Jurani CC et al. [275]	Yes	Yes	Yes	Yes	Yes	Yes	Yes	Yes	Include
de Belder MA et al. [276]	Yes	Yes	Yes	Yes	Yes	Yes	Yes	Yes	Include
Fiscon V et al. [277]	Yes	Yes	Yes	Yes	Yes	Yes	Unclear	Yes	Include
LoCicero J 3rd et al. [278]	Yes	Yes	Yes	Yes	Yes	Yes	Yes	Yes	Include
Bak YT et al. [279]	Yes	Yes	Yes	Yes	Yes	Yes	Yes	Yes	Include
Fiscon V et al. [280]	Yes	Yes	Yes	Yes	Yes	Yes	Yes	Yes	Include
Mittal S et al. [281]	Yes	Yes	Yes	Yes	Yes	Yes	Not applicable	Yes	Include
Walker WS et al. [282]	Yes	Yes	Yes	Yes	Yes	Yes	Yes	Yes	Include
Vernon N et al. [283]	Yes	Yes	Yes	Yes	Yes	Yes	Yes	Yes	Include
Saha A et al. [284]	Yes	Yes	Yes	Yes	Yes	Yes	Not applicable	Yes	Include
Sabanathan S et al. [285]	Yes	Yes	Yes	Yes	Yes	Yes	Yes	Yes	Include
Otsuka K et al. [286]	Yes	Yes	Yes	Yes	Yes	Yes	Yes	Yes	Include
Sakamoto Y et al. [287]	Yes	Yes	Yes	Yes	Yes	Yes	Yes	Yes	Include
Mahmodlou R et al. [288]	Yes	Yes	Yes	Yes	Yes	Yes	Yes	Yes	Include
Shinozuka E et al. [289]	Yes	Yes	Yes	Yes	Yes	Yes	Not applicable	Yes	Include
Ochiai T et al. [290]	Yes	Yes	Yes	Yes	Yes	Yes	Yes	Yes	Include
Kuwabara J et al. [291]	Yes	Yes	Yes	Yes	Yes	Yes	Yes	Yes	Include
Aga Z et al. [292]	Yes	Yes	Yes	Yes	Yes	Yes	Yes	Yes	Include
Berrisford RG et al. [293]	Yes	Yes	Yes	Yes	Yes	Yes	Unclear	Yes	Include
Kircheva DY et al. [294]	Yes	Yes	Yes	Yes	Yes	Yes	Unclear	Yes	Include
Ohri SK et al. [295]	Yes	Yes	Yes	Yes	Yes	Yes	Yes	Yes	Include
Petousis S et al. [296]	Yes	Yes	Yes	Yes	Yes	Yes	Yes	Yes	Include
Griffiths EA et al. [297]	Yes	Yes	Yes	Yes	Yes	Yes	Yes	Yes	Include

Hayakawa S et al. [298]	Yes	Yes	Yes	Yes	Yes	Yes	Not applicable	Yes	Include
Leoncini G et al. [299]	Yes	Yes	Yes	Yes	Yes	Yes	Yes	Yes	Include
Moriarity AR et al. [300]	Yes	Yes	Yes	Yes	Yes	Yes	Yes	Yes	Include
Eroğlu A et al. [301]	Yes	Yes	Yes	Yes	Yes	Yes	Not applicable	Yes	Include
Rustagi T et al. [302]	Yes	Yes	Yes	Yes	Yes	Yes	Not applicable	Yes	Include
Tanaka H et al. [165]	Yes	Yes	Yes	Yes	Yes	Yes	Yes	Yes	Include
Altorjay A et al. [304]	Yes	Yes	Yes	Yes	Yes	Yes	Yes	Yes	Include
Haws MJ et al. [305]	Yes	Yes	Yes	Yes	Yes	Yes	Yes	Yes	Include
Helton WB et al. [306]	Yes	Yes	Yes	Yes	Yes	Yes	Yes	Yes	Include
Altorjay A et al. [307]	Yes	Yes	Yes	Yes	Yes	Yes	Yes	Yes	Include
Nakashima S et al. [308]	Yes	Yes	Yes	Yes	Yes	Yes	Not applicable	Yes	Include
Larsson H et al. [309]	Yes	Yes	Yes	Yes	Yes	Yes	Yes	Yes	Include
Salminen P et al. [310]	Yes	Yes	Yes	Yes	Yes	Yes	Yes	Yes	Include
Gock M et al. [311]	Yes	Yes	Yes	Yes	Yes	Yes	Yes	Yes	Include
Hochberg LA et al. [312]	Yes	Yes	Yes	Yes	Yes	Yes	Unclear	Yes	Include
Wilde PH et al. [313]	Yes	Yes	Yes	Yes	Yes	Yes	Yes	Yes	Include
Larsson S et al. [314]	Yes	Yes	Yes	Yes	Yes	Yes	Yes	Yes	Include
Dye RA et al. [315]	Yes	Yes	Yes	Yes	Yes	Yes	Yes	Yes	Include
Bresadola V et al. [316]	Yes	Yes	Yes	Yes	Yes	Yes	Yes	Yes	Include
Morimoto Y et al. [317]	Yes	Yes	Yes	Yes	Yes	Yes	Unclear	Yes	Include
Mizutani K et al. [318]	Yes	Yes	Yes	Yes	Yes	Yes	Yes	Yes	Include
Clark DH et al. [319]	Yes	Yes	Yes	Yes	Yes	Yes	Not applicable	Yes	Include
Ghandour Z et al. [320]	Yes	Yes	Yes	Yes	Yes	Yes	Not applicable	Yes	Include
Cascio A et al. [321]	Yes	Yes	Yes	Yes	Yes	Yes	Yes	Yes	Include

Sidhu S et al. [322]	Yes	Yes	Yes	Yes	Yes	Yes	Not applicable	Yes	Include
Toghill PJ et al. [323]	Yes	Yes	Yes	Yes	Yes	Yes	Yes	Yes	Include
Wilson RF et al. [324]	Yes	Yes	Yes	Yes	Yes	Yes	Yes	Yes	Include
Harrington JD. [325]	Yes	Yes	Yes	Yes	Yes	Yes	Yes	Yes	Include
Drinkwater JB. [326]	Yes	Yes	Yes	Yes	Yes	Yes	Not applicable	Yes	Include
Chen TC et al. [327]	Yes	Yes	Yes	Yes	Yes	Yes	Yes	Yes	Include
Cochrane P. [328]	Yes	Yes	Yes	Yes	Yes	Yes	Yes	Yes	Include
Klygis LM et al. [329]	Yes	Yes	Yes	Yes	Yes	Yes	Yes	Yes	Include
Bradham RR et al. [330]	Yes	Yes	Yes	Yes	Yes	Yes	Not applicable	Yes	Include
Hamilton SG. [331]	Yes	Yes	Yes	Yes	Yes	Yes	Not applicable	Yes	Include
Limburg AJ et al. [332]	Yes	Yes	Yes	Yes	Yes	Yes	Unclear	Yes	Include
Mayer JE et al. [333]	Yes	Yes	Yes	Yes	Yes	Yes	Yes	Yes	Include
O'Connell ND. [334]	Yes	Yes	Yes	Yes	Yes	Yes	Yes	Yes	Include
Pandey CK et al. [335]	Yes	Yes	Yes	Yes	Yes	Yes	Yes	Yes	Include
Härmä RA et al. [336]	Yes	Yes	Yes	Yes	Yes	Yes	Yes	Yes	Include
Millard AH. [337]	Yes	Yes	Yes	Yes	Yes	Yes	Yes	Yes	Include
Katz SJ et al. [338]	Yes	Yes	Yes	Yes	Yes	Yes	Yes	Yes	Include
Dejode LR. [339]	Yes	Yes	Yes	Yes	Yes	Yes	Not applicable	Yes	Include
Phillipps JJ. [340]	Yes	Yes	Yes	Yes	Yes	Yes	Not applicable	Yes	Include
Sanderson RG. [341]	Yes	Yes	Yes	Yes	Yes	Yes	Unclear	Yes	Include
Chung CH. [342]	Yes	Yes	Yes	Yes	Yes	Yes	Yes	Yes	Include
Andersen OS et al. [343]	Yes	Yes	Yes	Yes	Yes	Yes	Yes	Yes	Include
Matsumoto MA et al. [344]	Yes	Yes	Yes	Yes	Yes	Yes	Unclear	Yes	Include
Asplund CM et al. [345]	Yes	Yes	Yes	Yes	Yes	Yes	Yes	Yes	Include

Becker GD. [346]	Yes	Yes	Yes	Yes	Yes	Yes	Unclear	Yes	Include
Hutter JA et al. [347]	Yes	Yes	Yes	Yes	Yes	Yes	Unclear	Yes	Include
Westaby S. [348]	Yes	Yes	Yes	Yes	Yes	Yes	Yes	Yes	Include
Case record of the Massachusetts General Hospital [349]	Yes	Yes	Yes	Yes	Yes	Yes	Not applicable	Yes	Include
Ando H et al. [350]	Yes	Yes	Yes	Yes	Yes	Yes	Not applicable	Yes	Include
Symbas PN et al. [351]	Yes	Yes	Yes	Yes	Yes	Yes	Yes	Yes	Include
Shaligram A et al. [352]	Yes	Yes	Yes	Yes	Yes	Yes	Yes	Yes	Include
McGovern M. [353]	Yes	Yes	Yes	Yes	Yes	Yes	Not applicable	Yes	Include
Ikeda Y et al. [354]	Yes	Yes	Yes	Yes	Yes	Yes	Not applicable	Yes	Include
Agrawal V et al. [355]	Yes	Yes	Yes	Yes	Yes	Yes	Yes	Yes	Include
Liang H et al. [356]	Yes	Yes	Yes	Yes	Yes	Yes	Yes	Yes	Include
Ersin OE et al. [357]	Yes	Yes	Yes	Yes	Yes	Yes	Yes	Yes	Include
Pei B et al. [358]	Yes	Yes	Yes	Yes	Yes	Yes	Yes	Yes	Include
Magalhães E et al. [359]	Yes	Yes	Yes	Yes	Yes	Yes	Unclear	Yes	Include
Soares C et al. [360]	Yes	Yes	Yes	Yes	Yes	Yes	Unclear	Yes	Include
Xiong SY et al. [361]	Yes	Yes	Yes	Yes	Yes	Yes	Yes	Yes	Include
Calderón-Cabrera DC et al. [362]	Yes	Yes	Yes	Yes	Yes	Yes	Not applicable	Yes	Include
Gharingam ML et al. [363]	Yes	Yes	Yes	Yes	Yes	Yes	Yes	Yes	Include
Bakhshi G et al. [364]	Yes	Yes	Yes	Yes	Yes	Yes	Yes	Yes	Include
Hussey T et al. [365]	Yes	Yes	Yes	Yes	Yes	Yes	Yes	Yes	Include
Chenevas-Paule Q et al. [366]	Yes	Yes	Yes	Yes	Yes	Yes	Not applicable	Yes	Include
Ishimine D et al. [367]	Yes	Yes	Yes	Yes	Yes	Yes	Yes	Yes	Include
Mendes I et al. [368]	Yes	Yes	Yes	Yes	Yes	Yes	Not applicable	Yes	Include

Han M et al. [369]	Yes	Yes	Yes	Yes	Not applicable	Not applicable	Not applicable	Yes	Include
Tzioti MK et al. [370]	Yes	Yes	Yes	Yes	Yes	Yes	Not applicable	Yes	Include
Kuthadi M et al. [371]	Yes	Yes	Yes	Yes	Yes	Yes	Yes	Yes	Include
Soussi D et al. [372]	Yes	Yes	Yes	Yes	Yes	Yes	Yes	Yes	Include
Schalet R et al. [373]	Yes	Yes	Yes	Yes	Yes	Yes	Yes	Yes	Include
Morgom M et al. [374]	Yes	Yes	Yes	Yes	Yes	Yes	Not applicable	Yes	Include
Eremia IA et al. [375]	Yes	Yes	Yes	Yes	Yes	Yes	Yes	Yes	Include
Mangeshkar S et al. [376]	Yes	Yes	Yes	Yes	Yes	Yes	Not applicable	Yes	Include
Ribeiro R et al. [377]	Yes	Yes	Yes	Yes	Yes	Yes	Yes	Yes	Include
Tipton KN et al. [378]	Yes	Yes	Yes	Yes	Yes	Yes	Yes	Yes	Include
Bhojani F et al. [379]	Yes	Yes	Yes	Yes	Yes	Yes	Yes	Yes	Include
Aldoseri R et al. [380]	Yes	Yes	Yes	Yes	Yes	Yes	Not applicable	Yes	Include
Qureshi A et al. [381]	Yes	Yes	Yes	Yes	Yes	Yes	Yes	Yes	Include
Nakano Y et al. [382]	Yes	Yes	Yes	Yes	Yes	Yes	Not applicable	Yes	Include
Mahant SS et al. [383]	Yes	Yes	Yes	Yes	Yes	Yes	Yes	Yes	Include
Redan JA et al. [384]	Yes	Yes	Yes	Yes	Yes	Yes	Not applicable	Yes	Include
Swied MY et al. [385]	Yes	Yes	Yes	Yes	Yes	Yes	Yes	Yes	Include
Ravikumar DB et al. [386]	Yes	Yes	Yes	Yes	Yes	Yes	Yes	Yes	Include
Ito S et al. [387]	Yes	Yes	Yes	Yes	Yes	Yes	Not applicable	Yes	Include
Estorninho J et al. [388]	Yes	Yes	Yes	Yes	Yes	Yes	Not applicable	Yes	Include
Kwiatkowska NM et al. [389]	Yes	Yes	Yes	Yes	Yes	Yes	Yes	Yes	Include
Totsi A et al. [390]	Yes	Yes	Yes	Yes	Yes	Yes	Yes	Yes	Include
Zhang J et al. [391]	Yes	Yes	Yes	Yes	Yes	Yes	Not applicable	Yes	Include
Huynh N et al. [392]	Yes	Yes	Yes	Yes	Yes	Yes	Yes	Yes	Include

Piratheepan A et al. [393]	Yes	Yes	Yes	Yes	Yes	Yes	Yes	Yes	Include
Parapar Álvarez L et al. [394]	Yes	Yes	Yes	Yes	Yes	Yes	Unclear	Yes	Include
Canelas Mendes C et al. [395]	Yes	Yes	Yes	Yes	Yes	Yes	Yes	Yes	Include
Shaik MR et al. [396]	Yes	Yes	Yes	Yes	Yes	Yes	Yes	Yes	Include
Tarazona MAD et al. [397]	Yes	Yes	Yes	Yes	Yes	Yes	Not applicable	Yes	Include
Jáquez-Quintana JO et al. [398]	Yes	Yes	Yes	Yes	Yes	Yes	Yes	Yes	Include
Samidoust P et al. [399]	Yes	Yes	Yes	Yes	Yes	Yes	Yes	Yes	Include
Loftus IA et al. [400]	Yes	Yes	Yes	Yes	Yes	Yes	Not applicable	Yes	Include
Darlington CR et al. [401]	Yes	Yes	Yes	Yes	Yes	Yes	Not applicable	Yes	Include
Pattynama LMD et al. [402]	Yes	Unclear	Yes	Yes	Yes	Yes	Yes	Yes	Include
Khadka B et al. [403]	Yes	Yes	Yes	Yes	Yes	Yes	Unclear	Yes	Include
Oh S et al. [404]	Yes	Yes	Yes	Yes	Yes	Yes	Unclear	Yes	Include
Ariza-Traslaviña J et al. [405]	Yes	Yes	Yes	Yes	Yes	Yes	Yes	Yes	Include
Tanaka T et al. [406]	Yes	Yes	Yes	Yes	Yes	Yes	Yes	Yes	Include
Maki H et al. [407]	Yes	Yes	Yes	Yes	Yes	Yes	Yes	Yes	Include
Bury J et al. [408]	Yes	Yes	Yes	Yes	Yes	Yes	Yes	Yes	Include
Nachiappan M et al. [409]	Yes	Yes	Yes	Yes	Yes	Yes	Not applicable	Yes	Include
Burnett TB et al. [410]	Yes	Yes	Yes	Yes	Yes	Yes	Yes	Yes	Include
Tan N et al. [411]	Yes	Yes	Yes	Yes	Yes	Yes	Yes	Yes	Include
Kita R et al. [412]	Yes	Yes	Yes	Yes	Yes	Yes	Yes	Yes	Include
Kakar N et al. [413]	Yes	Yes	Yes	Yes	Yes	Yes	Not applicable	Yes	Include
Anand R et al. [414]	Yes	Yes	Yes	Yes	Yes	Yes	Yes	Yes	Include
Matsuura N et al. [415]	Yes	Yes	Yes	Yes	Yes	Yes	Unclear	Yes	Include

Tanaka Y et al. [416]	Yes	Yes	Yes	Yes	Yes	Yes	Unclear	Yes	Include
Tuñon C et al. [417]	Yes	Yes	Yes	Yes	Yes	Yes	Yes	Yes	Include
Park SB et al. [418]	Yes	Yes	Yes	Yes	Yes	Yes	Yes	Yes	Include
Simões J et al. [419]	Yes	Yes	Yes	Yes	Yes	Yes	Yes	Yes	Include
Wang J et al. [420]	Yes	Yes	Yes	Yes	Yes	Yes	Unclear	Yes	Include
Chew FY et al. [421]	Yes	Yes	Yes	Yes	Yes	Yes	Unclear	Yes	Include
Gurgenidze M et al. [422]	Yes	Yes	Yes	Yes	Yes	Yes	Unclear	Yes	Include
Jagielski M et al. [423]	Yes	Yes	Yes	Yes	Yes	Yes	Unclear	Yes	Include
Śnieżyński J et al. [424]	Yes	Yes	Yes	Yes	Yes	Yes	Yes	Yes	Include
Inoue T et al. [425]	Yes	Yes	Yes	Yes	Yes	Yes	Unclear	Yes	Include
Zhu C et al. [426]	Yes	Yes	Yes	Yes	Yes	Yes	Yes	Yes	Include
Chen JJ et al. [427]	Yes	Yes	Yes	Yes	Yes	Yes	Unclear	Yes	Include
Jahangir A et al. [428]	Yes	Yes	Yes	Yes	Yes	Yes	Yes	Yes	Include
Zeyara A et al. [429]	Yes	Yes	Yes	Yes	Yes	Yes	Yes	Yes	Include
Abrahim M et al. [430]	Yes	Yes	Yes	Yes	Yes	Yes	Unclear	Yes	Include
Zimmer V. [84]	Yes	Yes	Yes	Yes	Yes	Yes	Unclear	Yes	Include
Chang G CW et al. [432]	Yes	Yes	Yes	Yes	Yes	Yes	Not applicable	Yes	Include
Amirian A et al. [433]	Yes	Yes	Yes	Yes	Yes	Yes	Yes	Yes	Include
Klug TD et al. [434]	Yes	Yes	Yes	Yes	Yes	Yes	Yes	Yes	Include
Hashmi MAR et al. [222]	Yes	Yes	Yes	Yes	Yes	Yes	Yes	Yes	Include
Awofisoye OI et al. [436]	Yes	Yes	Yes	Yes	Yes	Yes	Not applicable	Yes	Include
Ioannidis O et al. [437]	Yes	Yes	Yes	Yes	Yes	Yes	Yes	Yes	Include
Awadelkarim A et al. [438]	Yes	Yes	Yes	Yes	Yes	Yes	Yes	Yes	Include
Wang CT et al. [439]	Yes	Yes	Yes	Yes	Yes	Yes	Yes	Yes	Include

Ayazi K et al. [440]	Yes	Yes	Yes	Yes	Yes	Yes	Yes	Yes	Include
